# Protective effects of *Oxya chinensis sinuosa* Mishchenko against ultraviolet B-induced photodamage in hairless mice

**DOI:** 10.1186/s12906-019-2692-4

**Published:** 2019-10-28

**Authors:** A-Rang Im, InWha Park, Kon-Young Ji, Joo Young Lee, Ki Mo Kim, MinKyun Na, Sungwook Chae

**Affiliations:** 1 Herbal Medicine Research Division, Korea Institute of Oriental Medicine, Yuseong-daero 1672, Yuseong-gu, Daejeon, 34054 Republic of Korea; 2 Department of Pharmacognosy, College of Pharmacy, Chungnam National University, 99 Daehak-ro, Yuseong-gu, Daejeon, 34134 Republic of Korea; 3 University of Science and Technology, 217 Gajeong-ro, Yuseong-gu, Daejeon, 34113 Republic of Korea

**Keywords:** *Oxya chinensis sinuosa* Mishchenko, UVB irradiation, Matrix metalloproteinases, Mitogen-activated protein kinase, Photoprotective effect

## Abstract

**Background:**

Edible insects, including *Oxya chinensis sinuosa* Mishchenko (Oc), which is consumed as food in Asia, are considered as a human food shortage alternative, and also as a preventive measure against environmental destruction. Ultraviolet B (UVB) irradiation, which causes skin photodamage, is considered as an extrinsic skin aging factor. It reduces skin hydration, and increases wrinkle formation and reactive oxygen species (ROS) and inflammatory cytokine expression. Thus, the objective of this study was to investigate the anti-aging effects of an ethanol extract of Oc (Oc.Ex).

**Methods:**

A UVB-irradiated hairless mouse model was used to examine relevant changes in skin hydration, wrinkle formation, and skin epidermal thickness. Also, antioxidant markers such as superoxide dismutase (SOD) and catalase (CAT) were analyzed, and Oc. Ex skin protective effects against UVB irradiation-induced photoaging were examined by determining the levels of skin hydration factors.

**Results:**

Oc.Ex improved epidermal barrier dysfunctions such as increased transepidermal water loss (TEWL) and capacitance reduction in UVB-irradiated mice. It upregulated skin hydration-related markers, including hyaluronic acid (HA), transforming growth factor (TGF)-β, and pro-collagen, in UVB-irradiated mice, compared with the vehicle control group. It also reduced UVB-induced wrinkle formation, collagen degradation, and epidermal thickness. Additionally, it remarkably suppressed the increased expression of matrix metalloproteinases (MMPs), and restored the activity of SOD and CAT in UVB-irradiated mice, compared with the vehicle control group. Furthermore, Oc. Ex treatment downregulated the production of inflammatory cytokines and phosphorylation of the mitogen-activated protein kinases (MAPKs) signaling pathway activated by UVB irradiation.

**Conclusion:**

This study revealed that Oc. Ex reduced skin thickness and the degradation of collagen fibers by increasing hydration markers and collagen-regulating factors in the skin of UVB-irradiated mice. It also inhibited UVB-induced antioxidant enzyme activity and inflammatory cytokine expression via MAPK signaling downregulation, suggesting that it prevents UVB-induced skin damage and photoaging, and has potential for clinical development in skin disease treatment.

## Background

The process of skin aging can be classified as intrinsic or extrinsic aging [1]. Intrinsic aging is a natural process induced by metabolic, hormonal, and internal genetic factors, while extrinsic aging is induced by sun exposure, smoking, and environmental factors. Extrinsic aging induced by ultraviolet B (UVB) radiation causes skin alterations, including epidermal thickness, wrinkle formation, and matrix macromolecule degradation [2]. UVB irradiation qualitatively alters extracellular matrix (ECM) proteins, leading to collagen degradation induced by the upregulation of matrix metalloproteinases (MMPs) expression, and pro-collagen synthesis inhibition [3]. MMPs, which are known to play an important role in inflammation, cancer metastasis, and skin aging are enzymes that degrade skin ECM [4].

Skin dehydration is also involved in skin aging, and the principal skin moisture molecule hyaluronic acid (HA), also called hyaluronan or hyaluronate, is able to bind to and retain water molecules [5]. While skin moisture maintenance is essentially dependent on the stratum granulosum, HA binding with water is critically important in skin hydration retention in the dermis and the vital epidermis [6]. Also, the most widely used skin barrier function determination index is transepidermal water loss (TEWL) [7]. Thus, skin hydration maintenance is important in skin aging prevention.

Skin wrinkles are induced by skin elasticity reduction due to elastic fiber tortuosity and collagen fiber degradation [8]. UVB irradiation is known to increase the production of MMPs, thereby degrading the ECM and increasing wrinkles [9]. Reactive oxygen species (ROS) induces the secretion of MMPs from skin fibroblasts and keratinocytes, leading to collagen synthesis impairment, collagen and ECM proteins degradation, wrinkle formation, and skin photoaging [10]. The increased expression of MMP-1 due to increased mitogen-activated protein kinases (MAPKs) expression destroys skin tissue collagen matrix, thereby reducing skin elasticity and causing wrinkles [11].

UVB irradiation induces an increase in skin damage and inflammation, owing to the secretion of various cytokines such as interleukin (IL)-1, IL-6, and tumor necrosis factor (TNF)-α, which are cell-produced immune regulators [12]. Also, UVB-induced cell damage activates ROS-sensitive signaling molecules and pathways such as inflammatory cytokines and the MAPKs pathway [13], and increased ROS expression induced by UVB, causes inflammatory responses that promote skin aging.

Because of the increased concerns regarding global exhaustion of food supplies, due to the growing world population, insects have markedly attracted the interest of nutritional and toxicological fields [14]. Due to the increased need to identify and develop additional food and feed resources, owing to growing world population and the decreasing availability of arable land, insects are considered to be an important potential food source [15]. Thus, edible insects can serve as an excellent source of proteins and other nutrients. However, the possibility that the consumption of these edible insects could influence the human microbiome, due to the intake of the relatively understudied fiber source chitin, should also be considered [16]. Traditionally in Korea, *Oxya chinensis sinuosa* Mishchenko (Oc), belonging to the phylum Arthropoda (Order, 54 Orthoptera; Family, Acrididae; Subfamily, Oxyinae), is well known as the “famine relief insect” and was recently registered as a food in the Korean Food Standards Codex of the Ministry of Food & Drug Safety (MFDS) [17], and traditionally, it has been used to treat asthma, bronchitis, cough, whooping cough, paralysis, and seizures [18]. Although there is little information on treatment for various diseases, recent studies reported antiplatelet, antimicrobial effects of Oc [17, 18]. Moreover, our previous study showed its hepatoprotective effects in animal model of nonalcoholic fatty liver disease [19]. However, its ameliorating effects on photodamage and photoaging remains unclear. Thus, in this study, the potential protective effect of its ethanol extract (Oc.Ex) against UVB-induced skin damage was investigated.

## Methods

### Preparation of *Oxya chinensis sinuosa* Mishchenko extract

The material used in the preparation of the *Oxya chinensis sinuosa* Mishchenko extract, identified by Mi-Ae Kim, was obtained from the National Academy of Agricultural Science, Rural Development Administration (RDA), Korea. A voucher specimen (CNU-INS 201603) was deposited at the Pharmacognosy Laboratory of the College of Pharmacy, Chungnam National University, Daejeon, Korea. The *Oxya chinensis sinuosa* Mishchenko extract was prepared as previously reported [18], with a slight modification. Briefly, dried Oc (350 g, powder) was extracted with 70% ethanol (4 L) under reflux, and the extract was concentrated under vacuum to yield a brownish ethanol extract (47.87 g), which was lyophilized using a freeze-dryer (yield, approximately 13.68%).

### Experimental animals and oral administration

Male hairless mice (Hos/HR-1, 6 weeks old) purchased (Japan SLC, Inc., Sizuoka, Japan) and stabilized for 1 week before the study. The animals were housed under a 12 h/12 h light/dark cycle in a climate-controlled facility (temperature, 24 °C; humidity, 50%), with free access to food and water. All experiment protocols were approved by the Korea Institute of Oriental Medicine’s Institutional Animal Care and Use Committee (16–119). The mice were divided into three groups of six as follows: normal, UVB-irradiated vehicle, and UVB-irradiated Oc. Ex groups. Mice in the UVB-irradiated Oc. Ex group were orally administered 0.1 mL of water containing 100 mg Oc.Ex/kg of body weight per day. The normal group was neither irradiated with UVB nor administered any treatments.

### UVB irradiation

UVB irradiation was performed using a UVM-225D Mineralight UV Display Lamp (UVP, Phoenix, AZ, USA), which emitted radiation of wavelength 302 nm. The strength of the UV radiation was measured using a HD2102–2 UV meter (Delta OHM, Padova, Italy). It was applied on the backs of the mice three times per week for 12 weeks, and was progressively increased from 60 mJ/cm^2^ per exposure at week 1 (one minimal erythematous dose = 60 mJ/cm^2^) to 120 mJ/cm^2^ per exposure at week 12.

### Skin hydration and TEWL

A Corneometer and Tewameter (both from Courage+Khazaka electronic GmbH, Cologne, Germany) were used to measure skin hydration and TEWL, a marker of skin epidermis barrier function, respectively.

### Histological investigation

After the treatment period, animals were sacrificed using carbon dioxide aided asphyxiation, and after dissection, their dorsal skins were removed and fixed in 10% neutral-buffered formalin. Using a conventional method, the fixed tissues were washed, dehydrated, cleaned, infiltrated with and embedded in paraffin wax, and then 5 μm sections were cut and stained with hematoxylin and eosin (H&E) and Masson’s trichrome stain for collagen fiber analysis. Epidermis thickness was measured under a light microscope, using an eyepiece micrometer (Olympus Corporation, Tokyo, Japan).

### MMP-1, MMP-9, and HA secretion determination using enzyme-linked immunosorbent assay (ELISA)

MMP-1, MMP-9, and HA levels in skin tissue after UVB irradiation were determined using total MMP-1, MMP-9, and HA enzyme-linked immunosorbent assay (ELISA) kits, following the manufacturer’s instructions (R&D Systems, Minneapolis, MN, USA). MMP-1 and MMP-9 levels were then quantified via colorimetric analysis with a plate reader (Molecular Devices, Sunnyvale, CA, USA).

### Antioxidant enzyme activities

Superoxide dismutase (SOD) and catalase (CAT) activities were measured using a colorimetric assay kit (Cayman Chemical Co., Ann Arbor, MI, USA), following the manufacturer’s protocol. For protein extraction, skin tissue samples were homogenized in a cold lysis buffer, and absorbance was measured at 450 and 540 nm using a plate reader (Molecular Devices), to determine SOD and CAT activities, respectively.

### RNA extraction and quantitative real-time polymerase chain reaction

Total RNA was extracted from the skin tissue of UVB-irradiated mice using TRIzol reagent (Invitrogen, Carlsbad, CA, USA), following the manufacturer’s protocol. Quantitative real-time polymerase chain reaction (qRT-PCR) was performed using TaqMan assay kits (Applied Biosystems, Foster City, CA, USA) specific for transforming growth factor (TGF)-β, Mm00436960_m1; IL-1β, Mm00434228_m1; IL-6, Mm00446190_m1; and TNF-α, Mm00443258_m1, using a QuantStudio™ 6 Flex real-time PCR system (Applied Biosystems). Each sample was assayed in triplicates, and the relative mRNA expression levels in each sample were calculated using the ΔΔCt method and normalized to β-actin mRNA levels.

### Western blotting

Protein was extracted from the skin tissue samples, and the protein lysates (20 μg) of each sample were electrophoresed on a 10% sodium dodecyl sulfate-polyacrylamide gel, and then transferred onto polyvinylidene fluoride membranes, which were then blocked with a 5% blocking solution (ATTO, Tokyo, Japan) for 1 h at room temperature. After the blocking, the blots were incubated overnight at 4 °C with a monoclonal antibody (1:1000), thereafter, they were washed three times for 10 min each in Tris-buffered saline (TBS), and then incubated for 2 h with a secondary antibody, and the proteins were detected using an enhanced chemiluminescence solution, with an LAS-4000 mini luminescent image analyzer (Fujifilm, Dusseldorf, Germany) (Additional file 1).

### Statistical analyses

All measurements were performed in triplicates, and the data are presented as mean ± standard error (SE). Analysis of variance (ANOVA) with the Tukey’s test was used to analyze group differences, and *p* < 0.05 was considered statistically significant.

## Results

### Evaluation of *Oxya chinensis sinuosa* Mishchenko extract on skin hydration factors

**Fig. 1:**
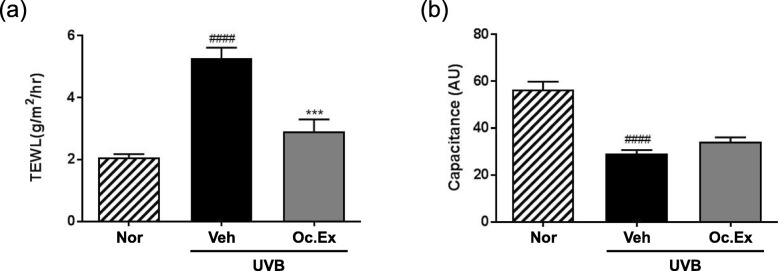
Effects of *Oxya chinensis sinuosa* Mishchenko extract (Oc.Ex) on ultraviolet B (UVB)-induced skin hydration. **a** Transepidermal water loss (TEWL) and **b** capacitance in UVB-irradiated hairless mice after > 10 weeks Oc. Ex administration. Data are compared with those of the normal (^####^*p* < 0.0001) and vehicle (***p < 0.001) groups. Nor, Normal; Veh, Vehicle; Oc. Ex, *Oxya chinensis sinuosa* Mishchenko extract

**Fig. 2:**
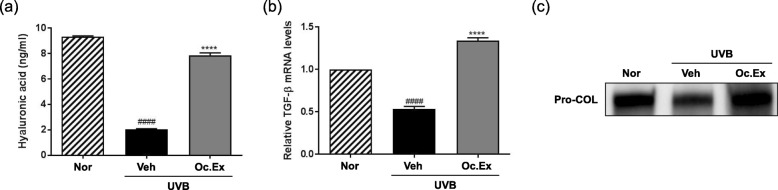
Effects of *Oxya chinensis sinuosa* Mishchenko extract (Oc.Ex) on ultraviolet B (UVB)-induced skin hydration. **a** Hyaluronic acid (HA), **b** transforming growth factor (TGF)-β mRNA, and **c** pro-collagen expression levels in skin tissue. Data are compared with those of the normal (^####^*p* < 0.0001) and vehicle (****p < 0.0001) groups. Nor, Normal; Veh, Vehicle; Oc. Ex, *Oxya chinensis sinuosa* Mishchenko extract

To determine whether Oc. Ex regulated skin hydration level, the TEWL and capacitance of UVB-irradiated hairless mice skin were evaluated. The results revealed that in the UVB-irradiated vehicle group, TEWL levels, which recovered after Oc. Ex treatment, were significantly higher than those in the normal group (Fig. 1a). Also, their capacitance decreased compared with the normal mice group, and slightly recovered after Oc. Ex treatment (Fig. 1b). Additionally, analysis of the effect of Oc. Ex on skin hydration-related markers, including HA content and TGF-β mRNA levels, using an assay kit and RT-PCR, respectively, revealed that Oc. Ex treatment improved HA content and TGF-β mRNA levels, which were suppressed by UVB irradiation (Fig. 2a and b). Further, skin tissue pro-collagen expression, which was decreased by UVB-irradiated, increased in the Oc.Ex-treated group (Fig. 2c). Thus, Oc. Ex reduces UVB irradiation-induced skin damage, which is associated with decreased TEWL and increased skin hydration.

### Histology of the anti-wrinkle effect of *Oxya chinensis sinuosa* Mishchenko extract on UVB-irradiated hairless mice

**Fig. 3:**
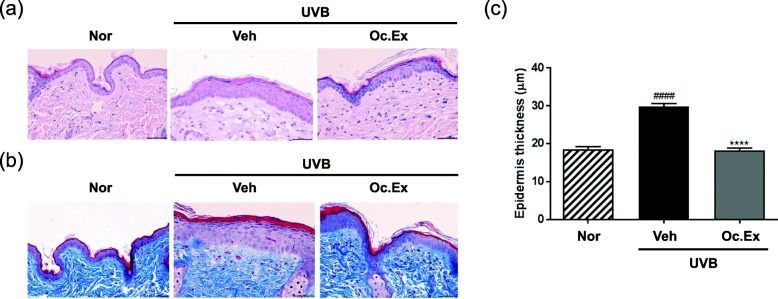
Effect of *Oxya chinensis sinuosa* Mishchenko extract (Oc.Ex) on ultraviolet B (UVB)-induced skin thickening in hairless mice. **a** Hematoxylin and eosin (H&E) staining of UVB-irradiated hairless mice skin (Original magnification, × 400). **b** Protective effect of Oc. Ex on collagen fiber changes (Histological observation of hairless mouse skin using Masson’s trichrome staining). Collagen fibers were stained blue, and images were obtained at × 400 magnification. **c** Dorsal skin epidermal thickness (Original magnification, × 400). Data are compared with those of the normal group (^####^*p* < 0.0001), and the percentages of the vehicle group (****p < 0.0001). Scale bar, 20 μm. Nor, Normal; Veh, Vehicle; Oc. Ex, *Oxya chinensis sinuosa* Mishchenko extract

To evaluate the anti-wrinkle effects of Oc. Ex on the skin of UVB-irradiated hairless mice, skin histological analysis was performed using H&E and Masson’s trichrome staining. Stratum corneum and epidermis depths in the skin of the vehicle treated group increased as a result of UVB irradiation, compared with the normal group, and Oc. Ex treatment led to their restoration (Fig. 3a). Also, the uniformly distributed collagen fibers in the skin of mice from the vehicle group were detracted by UVB irradiation, but improved by Oc. Ex treatment (Fig. 3b). To further evaluate the anti-wrinkle effect of Oc. Ex, thickness changes between the keratin layer and the epidermal basement membrane of the skin were analyzed via microscopic measurements in H&E stained skin. The epidermal thickness in the skin of the UVB-irradiated vehicle group, which was markedly decreased to normal by Oc. Ex treatment, was greater than that in the normal group (Fig. 3c). These results suggest that Oc. Ex exhibits an anti-wrinkle effect by reducing the increased thickness of stratum corneum and epidermis, and the impaired distribution of collagen fibers, induced by UVB irradiation.

### *Oxya chinensis sinuosa* Mishchenko extract inhibits UVB-induced MMPs expression

**Fig. 4:**
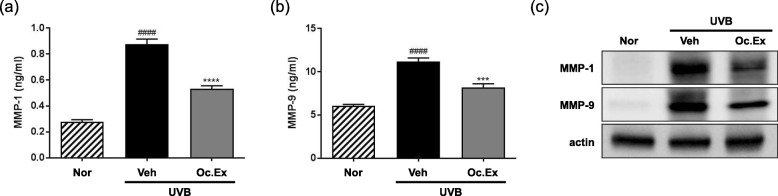
Effect of *Oxya chinensis sinuosa* Mishchenko extract (Oc.Ex) on matrix metalloproteinase (MMP) and procollagen expression. **a** MMP-1 and **b** MMP-9 protein levels in UVB-irradiated skin. **c** Western blotting of Oc. Ex effects on ultraviolet B (UVB)-mediated induction of MMP-1 and MMP-9. Data are compared with those of the normal (^####^*p* < 0.0001) and vehicle (****p < 0.0001 and ***p < 0.001) groups. Nor, Normal; Veh, Vehicle; Oc. Ex, *Oxya chinensis sinuosa* Mishchenko extract

To determine the inhibitory effect of Oc. Ex against UVB-induced MMP expression, MMP-1 and MMP-9 protein levels were analyzed using ELISA and western blotting, respectively. The analyses revealed that MMP-1 and MMP-9 expressions in the skin of hairless mice were upregulated by UVB irradiation, while Oc. Ex treatment downregulated their expression (Fig. 4a and b). Also, as shown in Fig. 4c, the protein expression of MMP-1 and MMP-9 was downregulated by Oc. Ex treatment.

### Effects of *Oxya chinensis sinuosa* Mishchenko extract on antioxidant enzymes in UVB-irradiated hairless mice

**Fig. 5:**
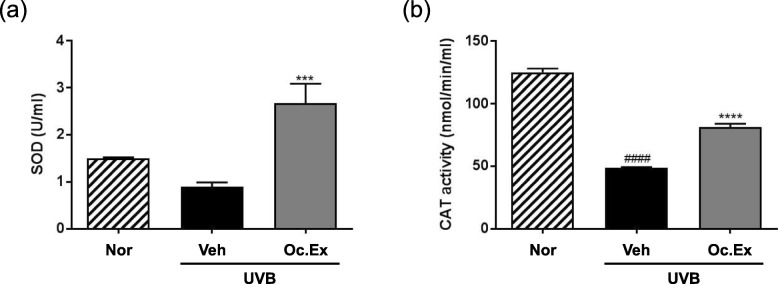
Effects of *Oxya chinensis sinuosa* Mishchenko extract (Oc.Ex) on antioxidant activity of **a** superoxide dismutase (SOD) and **b** catalase (CAT) in hairless mice skin exposed to ultraviolet B (UVB). Data are compared with those of the normal (^####^*p* < 0.0001) and vehicle (****p < 0.0001 and ***p < 0.001) groups. Nor, Normal; Veh, Vehicle; Oc. Ex, *Oxya chinensis sinuosa* Mishchenko extract

To investigate the inhibitory effect of Oc. Ex on free radical-scavenging activity via the regulation of antioxidant enzymes, SOD and CAT activities in the skin of hairless mice after UVB exposure were measured. SOD activity in the UVB-irradiated vehicle group was lower than that in the normal group, and was increased by Oc. Ex treatment (Fig. 5a). Also, CAT activity in the UVB-irradiated vehicle group was lower than that in the normal group, but was increased by Oc. Ex treatment (Fig. 5b).

### Effects of *Oxya chinensis sinuosa* Mishchenko extract on the mRNA expression of inflammatory cytokines

**Fig. 6:**
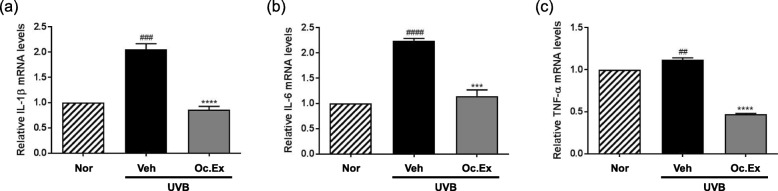
Effects of *Oxya chinensis sinuosa* Mishchenko extract (Oc.Ex) on pro-inflammatory cytokines in ultraviolet B (UVB)-irradiated hairless mouse skin. mRNA expression levels of **a** IL-1β **b** IL-6, and **c** TNF-α were determined via quantitative reverse transcription polymerase chain reaction. Data are compared with those of the normal (^####^*p* < 0.0001, ^###^*p* < 0.001 and ^##^*p* < 0.01) and vehicle (****p < 0.0001, and ***p < 0.001) groups. Nor, Normal; Veh, Vehicle; Oc. Ex, *Oxya chinensis sinuosa* Mishchenko extract

To verify the lowering effect of Oc. Ex on inflammatory cytokine levels increased by UVB irradiation, the mRNA expression of IL-1β, IL-6, and TNF-α were analyzed using qRT-PCR. As shown in Fig. 6, UVB irradiation significantly increased IL-1β, IL-6, and TNF-α gene expressions, which were remarkably restored by Oc. Ex treatment, suggesting that Oc. Ex exhibits a photoprotective effect by lowering UVB irradiation-induced inflammatory responses.

### Effect of *Oxya chinensis sinuosa* Mishchenko extract on the phosphorylation of MAPKs in UVB-irradiated hairless mice

**Fig. 7:**
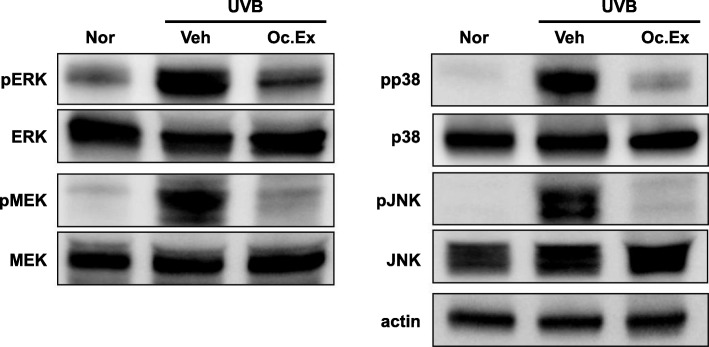
Effects of *Oxya chinensis sinuosa* Mishchenko extract (Oc.Ex) on the phosphorylation of mitogen-activated protein kinases (MAPKs) in an ultraviolet B (UVB)-irradiated mouse model. Oc. Ex inhibited MEK, ERK, p38, and JNK phosphorylation. Nor, Normal; Veh, Vehicle; Oc. Ex, *Oxya chinensis sinuosa* Mishchenko extract

MMP-9 production is primarily regulated by the activation of the MAPKs signaling pathway. Thus, the regulatory effect of Oc. Ex on the phosphorylation of the MAPKs signaling pathway in the skin of UVB-irradiated mice was examined. As shown in Fig. 7, there was no difference in total protein expression of extracellular signal-regulated kinase (ERK), MEK, p38, and c-Jun N-terminal kinase (JNK). Also, UVB irradiation increased the phosphorylation of ERK, MEK, p38, and JNK in the UVB-irradiated vehicle group compared with the normal group. Interestingly, this increased phosphorylation was markedly suppressed by Oc. Ex treatment.

## Discussion

This study, which aimed to evaluate the protective effect of *Oxya chinensis sinuosa* Mishchenko (Oc.Ex) against UVB-induced photodamage, demonstrated that Oc. Ex attenuated UVB-induced epidermal barrier dysfunction by increasing skin hydration (HA and TGF-β) as well as collagen-regulating factors (MMP-1, MMP-9, and pro-collagen). Also, UVB-induced antioxidant enzyme activity (SOD and CAT) and inflammatory cytokine expression (IL-1β, IL-6, and TNF-α) were suppressed by Oc. Ex treatment via downregulation of MAPK signaling transduction, suggesting that Oc. Ex prevents UVB-induced photodamage and has a therapeutic potential for skin disease treatment.

Photoaging skin exhibits distinct alterations, including coarse and deep wrinkle formation, thickened and leathery appearance, and irregular pigmentation [20], and these morphological alterations are often induced by chronic UVB irradiation [21]. Skin exposure to UVB, a minor but highly carcinogenic component of sunlight, induces several biological responses, including inflammation, systemic immunosuppression, erythema, hyperpigmentation, hyperplasia, and skin cancer [22].

Chronic exposure to UVB results in skin aging, which causes wrinkle formation, acute erythema, and loss of hydration and elasticity [23]. The index widely used to evaluate skin barrier integrity is TEWL and skin capacitance [24]. A low TEWL and a high capacitance are associated with skin barrier integrity, whereas the contrary indicates barrier disruption [25]. HA plays important roles in healthy skin by regulating epithelial cell phenotype, and it regulates general skin functions, including water retention, turgidity, elasticity, and nutrient diffusion [26]. Also, TGF-β critically regulates collagen homeostasis through pro-collagen I and III stimulation, and MMP-1 transcription inhibition [27]. This study demonstrated that a high TEWL value and low capacitance value were associated with UVB-irradiated skin, whereas Oc. Ex administration improved skin barrier function and restored the UVB irradiation-induced decrease in the expression of HA, TGF-β, and pro-collagen.

The distinctive features of photoaging are collagen degradation and abnormal elastin accumulation in the superficial dermis, and a number of MMPs are reported to be involved in these processes [28]. Exposure to UV radiation frequently aggravates wrinkle formation, skin pigmentation, and stimulates the expression and activation of MMPs, which are well known to play a leading role in the damage of skin connective tissues, due to collagen degradation and collagen synthesis inhibition [29]. Consistent with these previous studies, this study revealed that Oc. Ex treatment remarkably suppressed the expression of MMP-1 and MMP-9.

UV-induced oxidative stress upregulates ROS production by counteracting the activities of endogenous antioxidants, including SOD, CAT, and glutathione (GSH), which neutralize ROS before cell changes in oxidative production [30]. Skin oxidative stress is closely associated with UV irradiation-induced skin damage, and SOD and CAT are critical enzymes in oxidative stress [31]. Likewise, this study revealed that the antioxidant activity of skin tissue exposed to UVB irradiation can be determined by estimating SOD and CAT levels.

UV-induced ROS causes the oxidative damage of DNA, proteins, and cell membrane lipids in the exposed cells, and increases the expression of collagen, elastin, and HA degrading enzymes [32]. ROS production initiates UVB-induced photoaging, and activates a number of receptors such as IL-1, TNF-α, and keratinocyte growth factor, in epidermal keratinocytes [33]. In the present study, Oc. Ex treatment reduced the mRNA expression levels of IL-1β, IL-6, and TNF-α in the skin of UVB-irradiated hairless mice, establishing its inhibitory effect on the increased production of UVB-induced pro-inflammatory cytokines.

Finally, this study demonstrated the involvement of MAPKs in the antioxidant effect of Oc. Ex against UVB-induced oxidative stress. The MAPKs pathway, which includes molecules such as MEK, ERK, p38, and JNK, is upregulated by the response to extracellular stimuli such as UV irradiation [34]. To elucidate the pathway by which Oc. Ex mediates its photoprotective effect, its effects on the ERK, MEK, p38, and JNK MAPKs pathways were evaluated.

## Conclusions

To conclude, this study reports the photoprotective effect of Oc. Ex against skin damage, using UVB-induced hairless mice. It revealed that Oc. Ex inhibited UVB-induced skin thickening and wrinkle formation, and increased skin hydration factors and antioxidant enzymes in hairless mice. Further, Oc. Ex treatment attenuated UVB-induced MMPs, pro-inflammatory cytokines, and MAPK phosphorylation, suggesting that it prevents UVB-induced skin damage and photoaging.

## Supplementary information

**Additional file 1.** Uncropped images from the western blot data presented in main figures.

## Data Availability

The datasets used and/or analyzed during the current study are available from the corresponding author on reasonable request.

## Abbreviations

CAT: Catalase
HA: Hyaluronic acid
MAPKs: Mitogen-activated protein kinases
MMPs: Matrix metalloproteinases
Oc: *Oxya chinensis sinuosa* Mishchenko
ROS: Reactive oxygen species
SOD: Superoxide dismutase
TEWL: Transepidermal water loss
TGF-β: Transforming growth factor-beta
TNF-α: Tumor necrosis factor-α
UVB: Ultraviolet B
