# Supplementary figures and images for: Protective effects of Oxya chinensis sinuosa Mishchenko against ultraviolet B-induced photodamage in hairless mice
Source: BMC Complement Altern Med. 2019 Oct 28;19:286. doi: 10.1186/s12906-019-2692-4 (PMC6819654; doi:10.1186/s12906-019-2692-4)

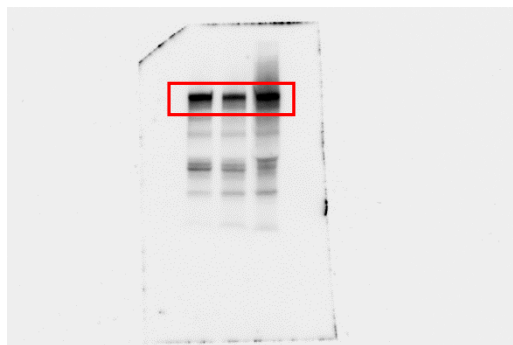

**proCOL**

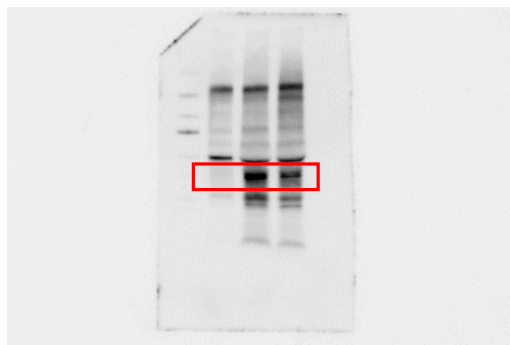

**MMP-1**

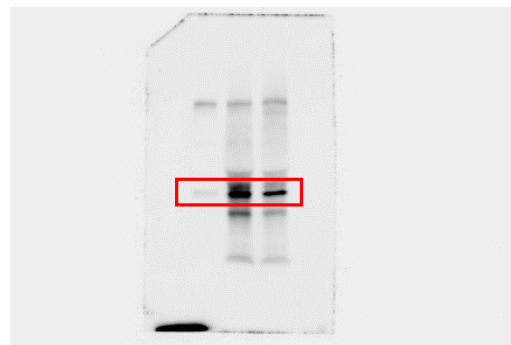

**MMP-9**

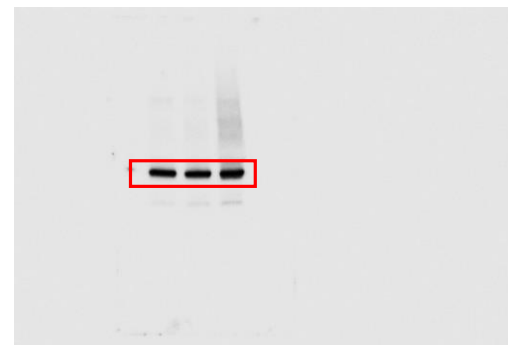

**actin**

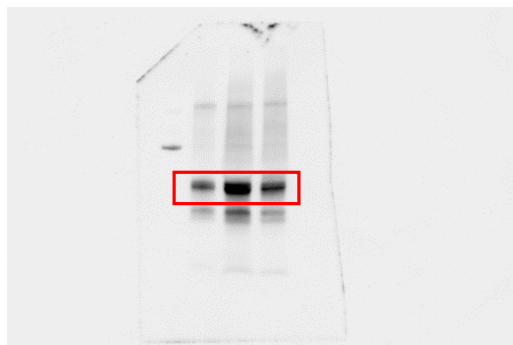

**pERK**

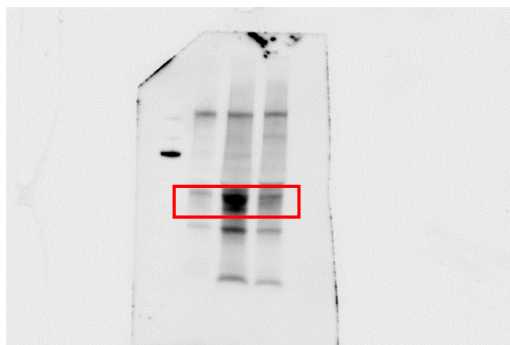

**pMEK**

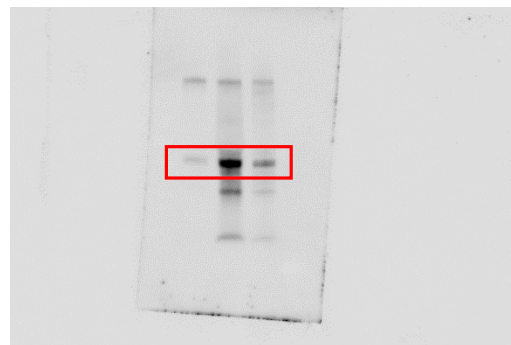

**pp38**

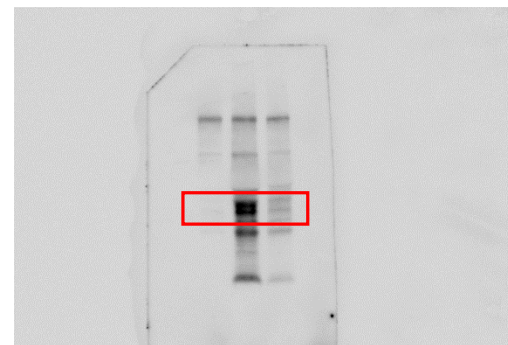

**pJNK**

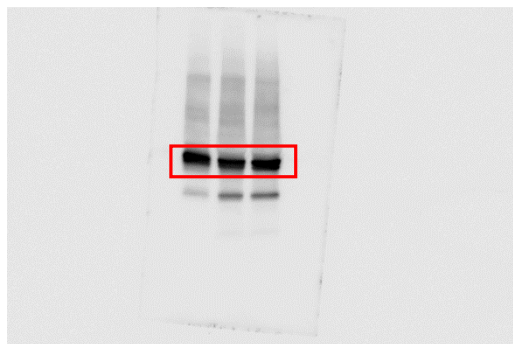

**ERK**

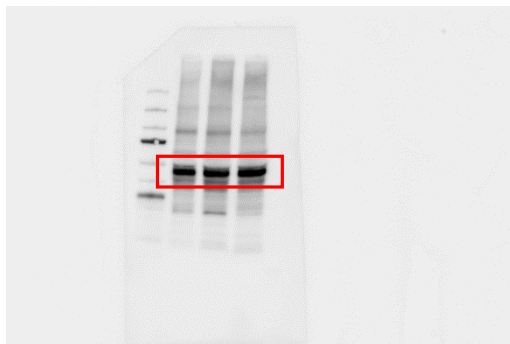

**MEK**

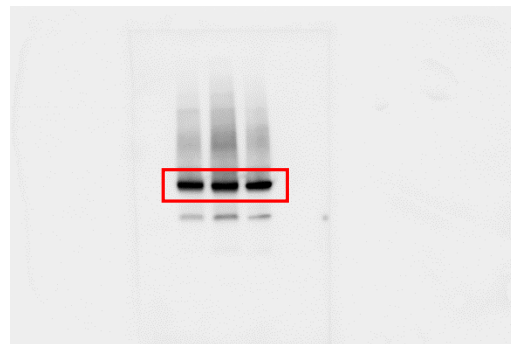

**p38**

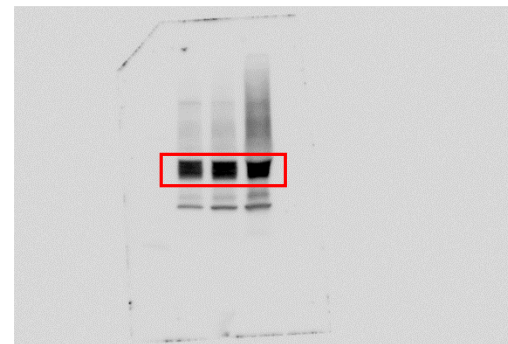

**JNK**

Supplement: Supplementary file 1 — Additional file 1. Uncropped images from the western blot data presented in main figures. [file 12906_2019_2692_MOESM1_ESM.pdf]
